# Supplementary material for: Establishment of reference intervals of clinical chemistry analytes for the adult population in Egypt
Source: PLoS One. 2021 Mar 19;16(3):e0236772. doi: 10.1371/journal.pone.0236772 (PMC7979267; doi:10.1371/journal.pone.0236772)

Suppl. Figure 1   Comparison of panel test results from two testing centers and assigned values

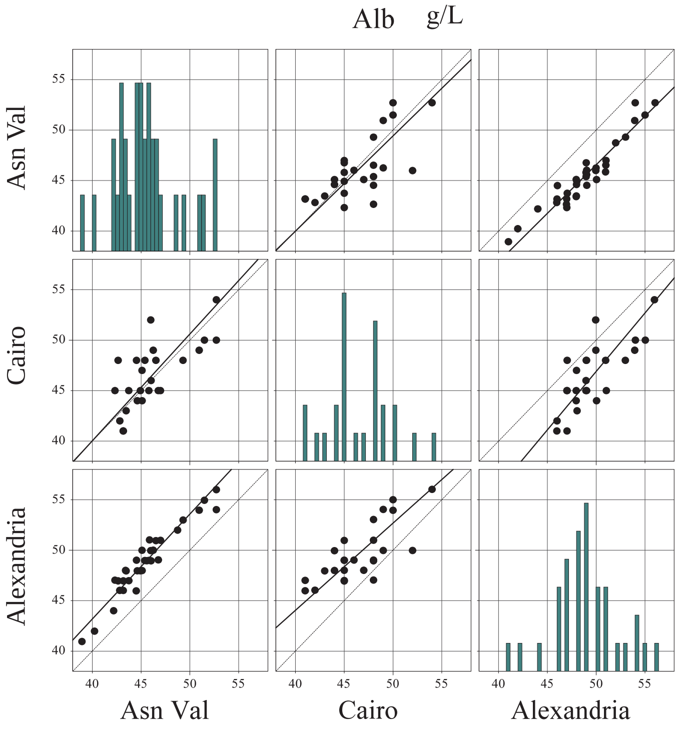

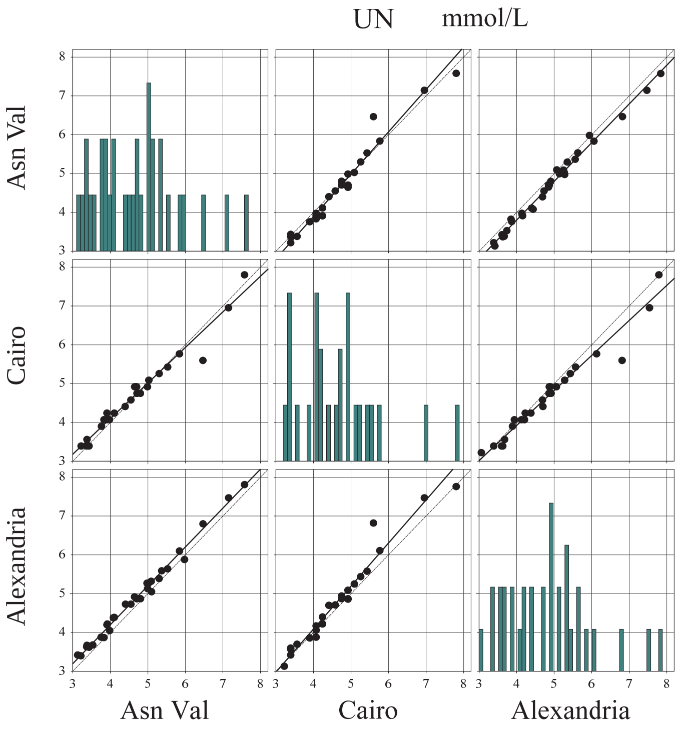

Asn Val = assigned value to the panel of sera

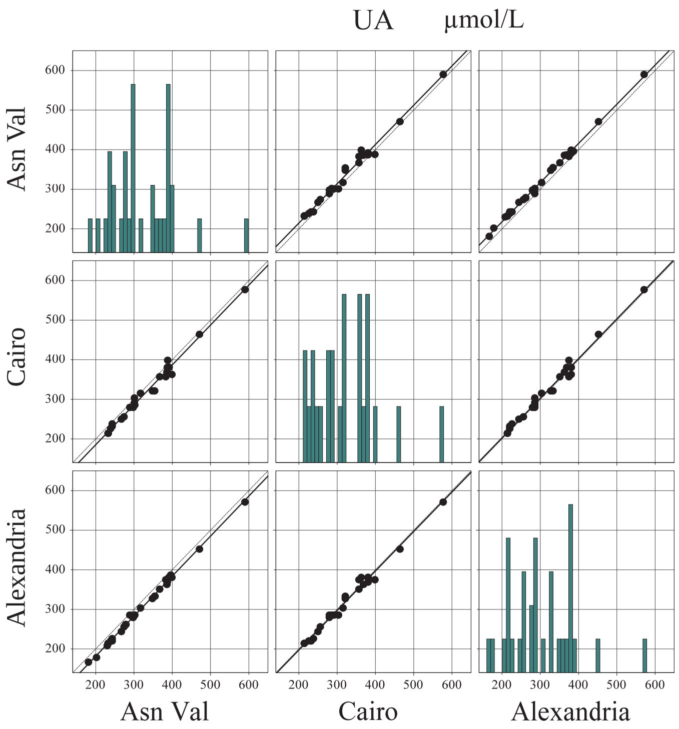

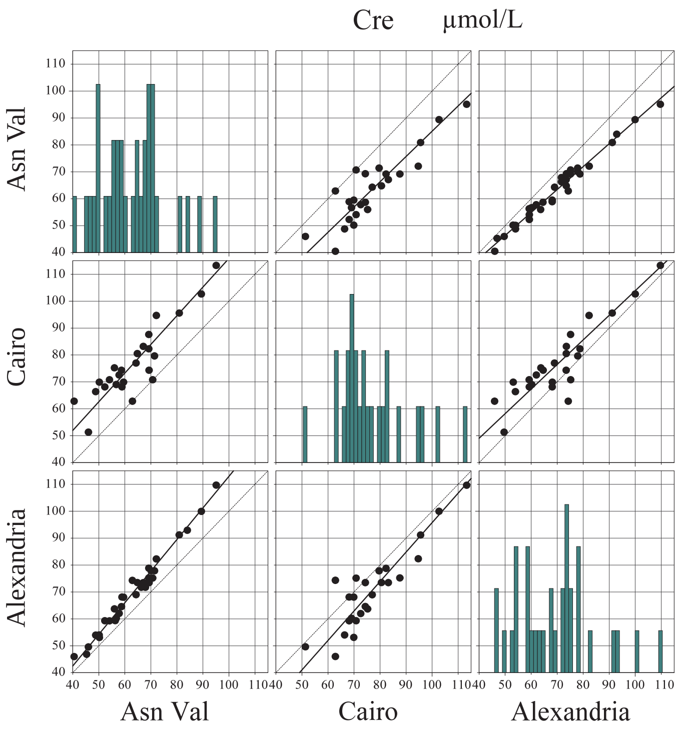

Na mmol/L

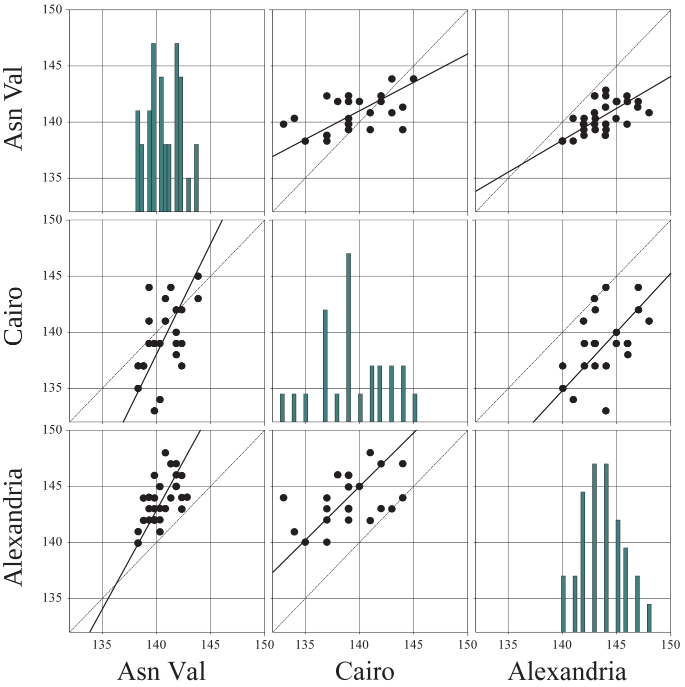

K mmol/L

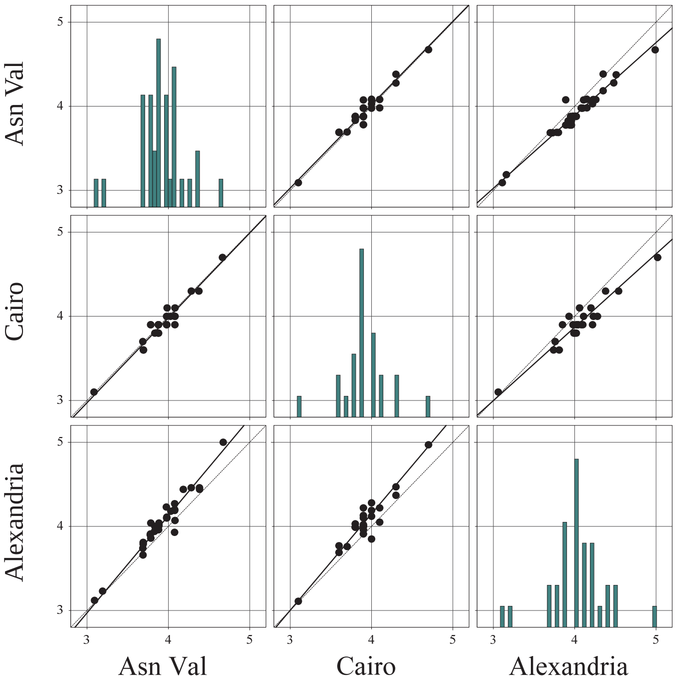

Cl mmol/L

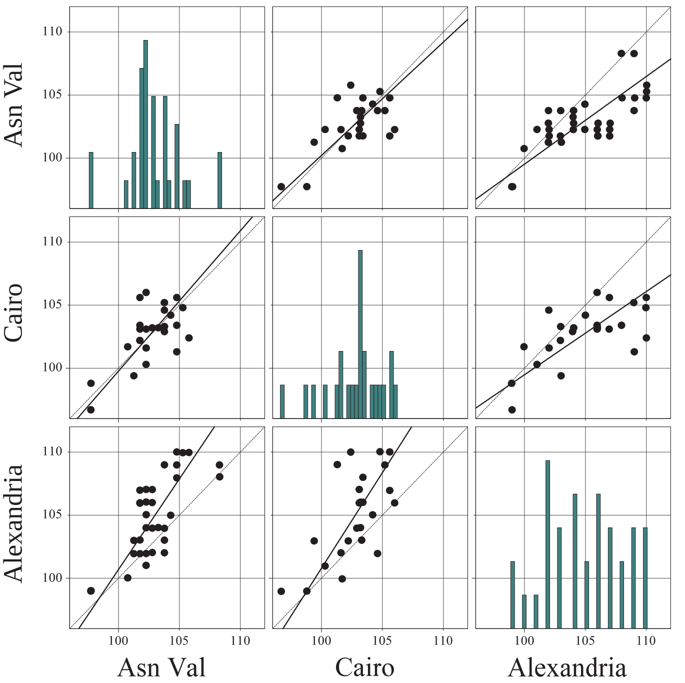

Ca mmol/L

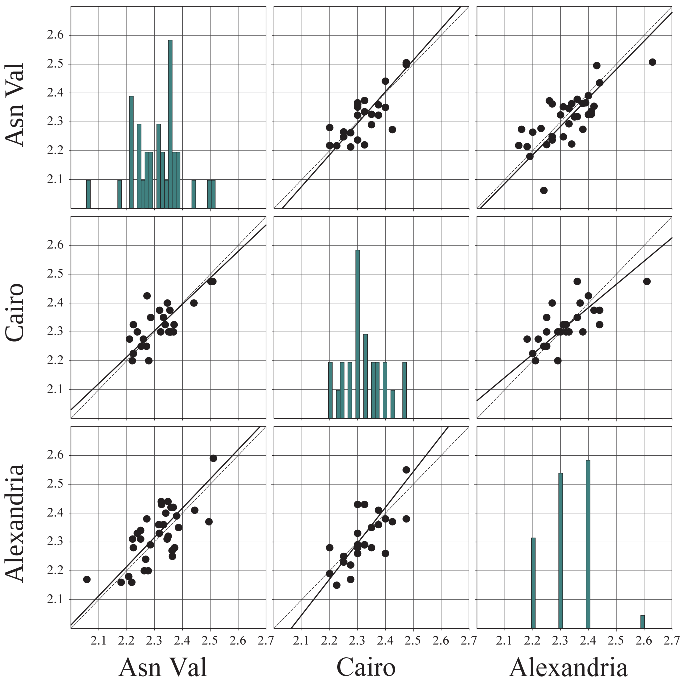

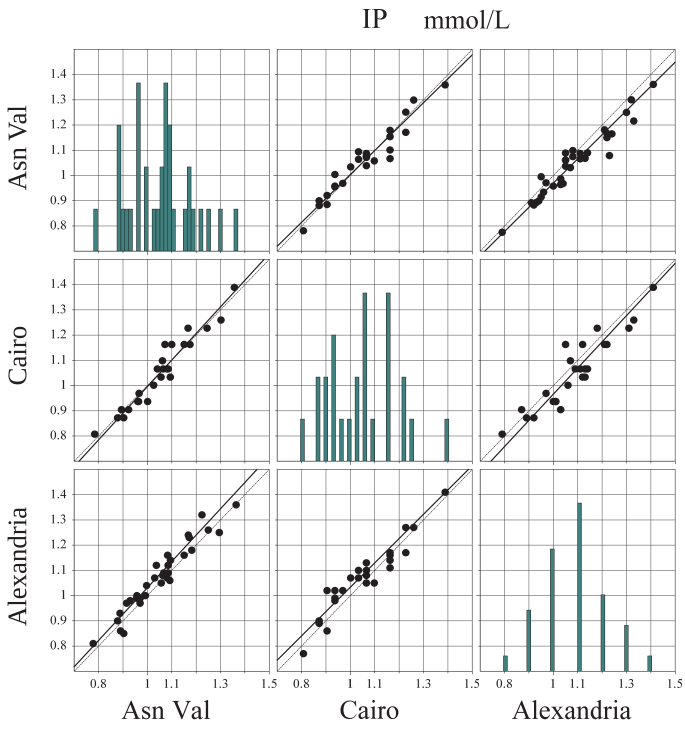

IP mmol/L
Asn Val
Cairo
Alexandria
Asn Val
Cairo
Alexandria

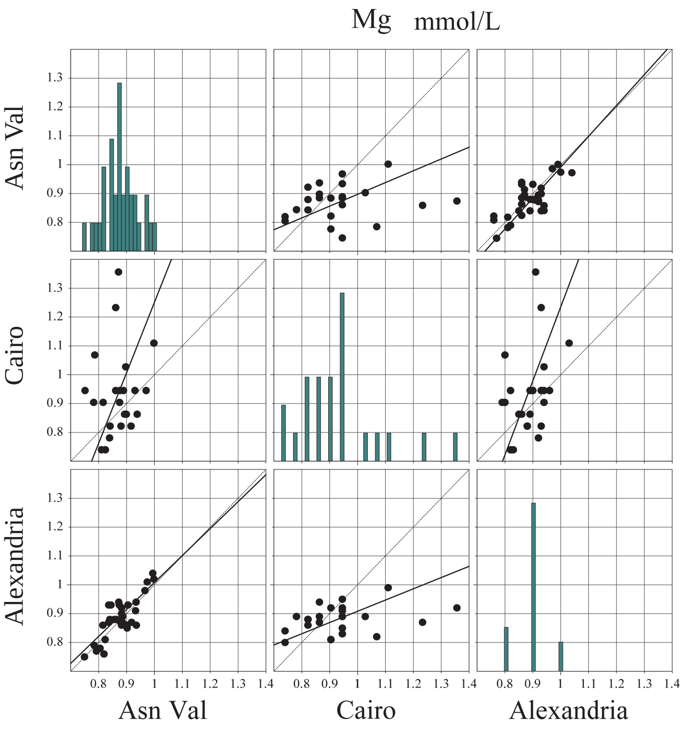

Mg mmol/L
Asn Val
Cairo
Alexandria
Asn Val
Cairo
Alexandria

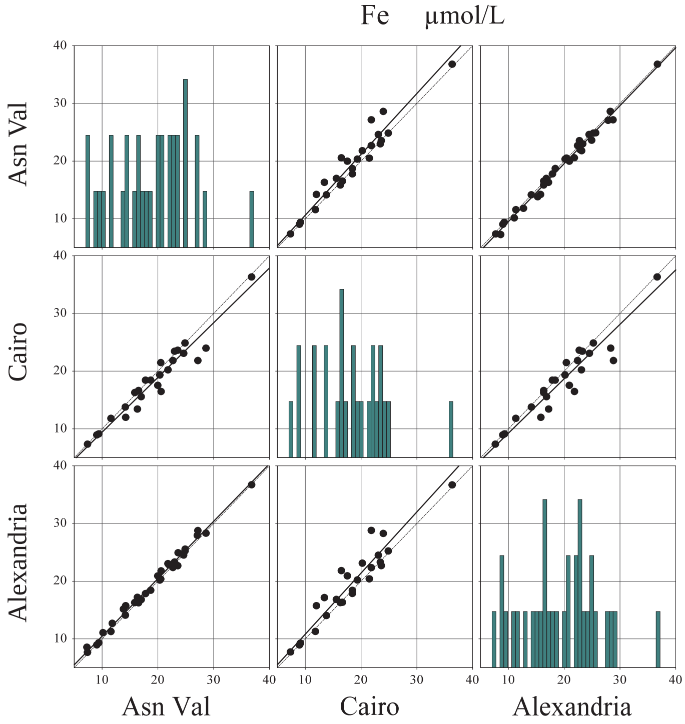

Fe µmol/L
Asn Val
Cairo
Alexandria
Asn Val
Cairo
Alexandria

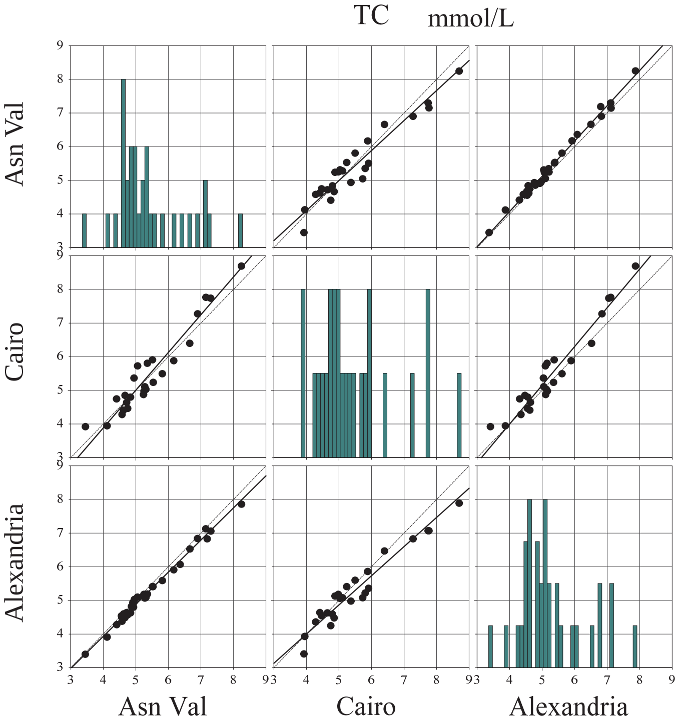

TC mmol/L
Asn Val
Cairo
Alexandria
Asn Val
Cairo
Alexandria

TG mmol/L

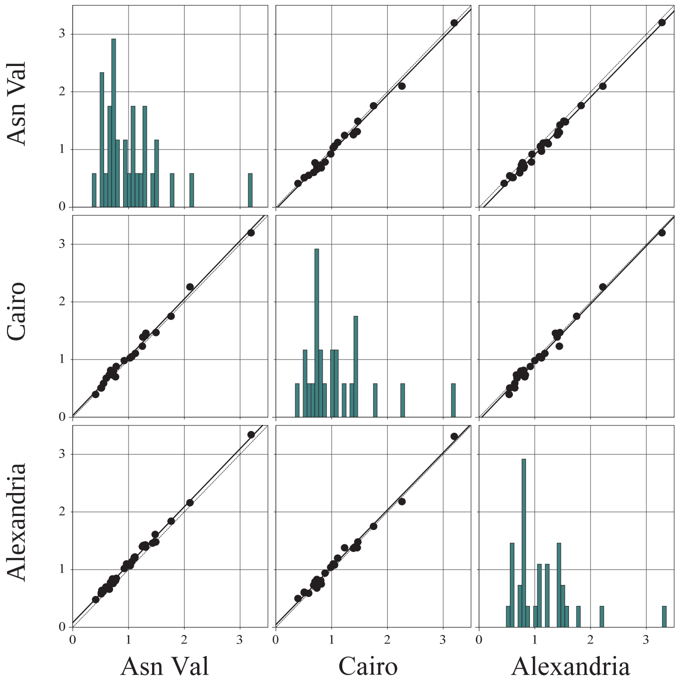

HDL-C mmol/L

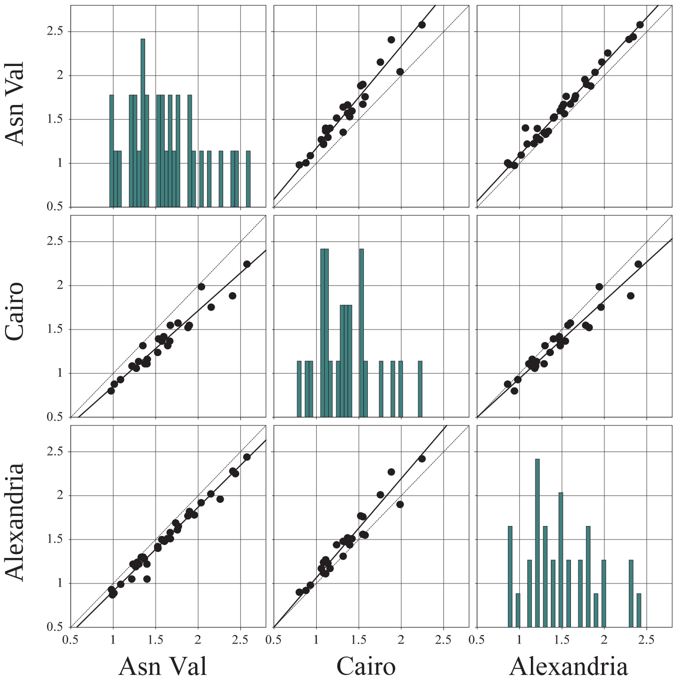

LDL-C mmol/L

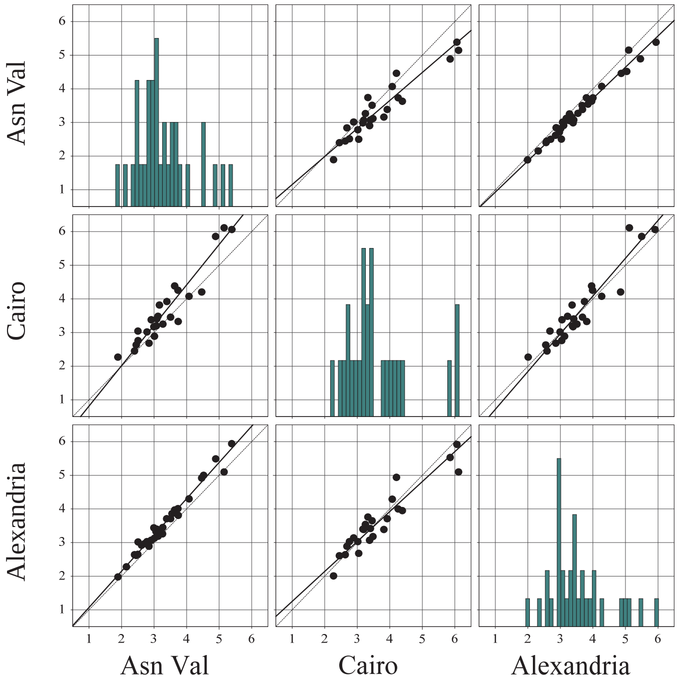

AST U/L

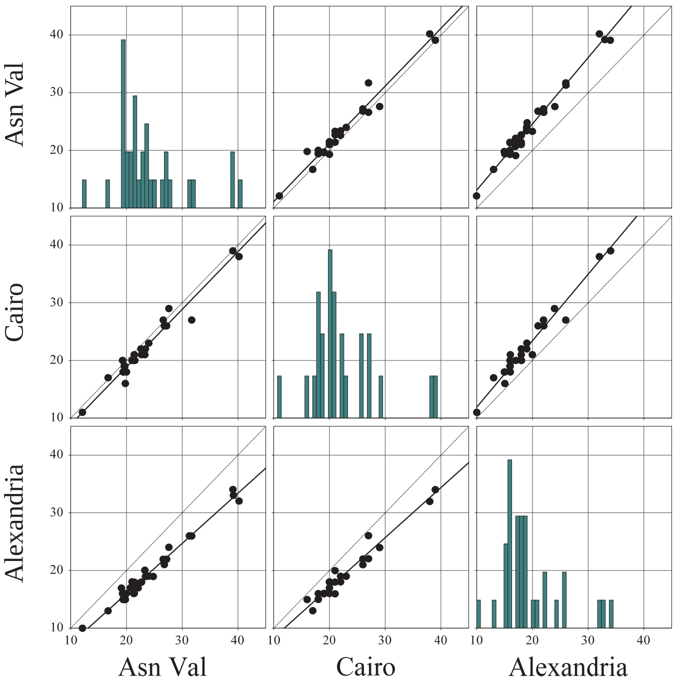

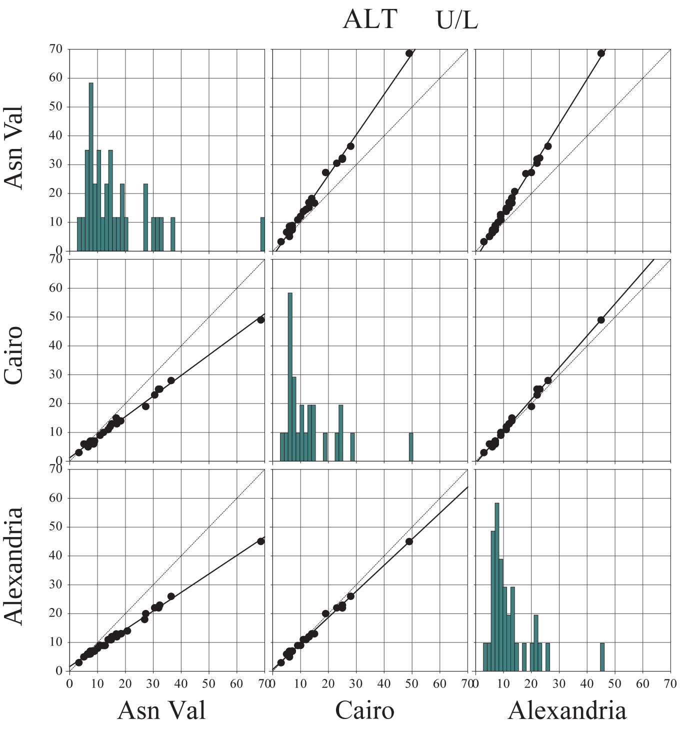
ALT U/L
Asn Val
Cairo
Alexandria
0 10 20 30 40 50 60 70

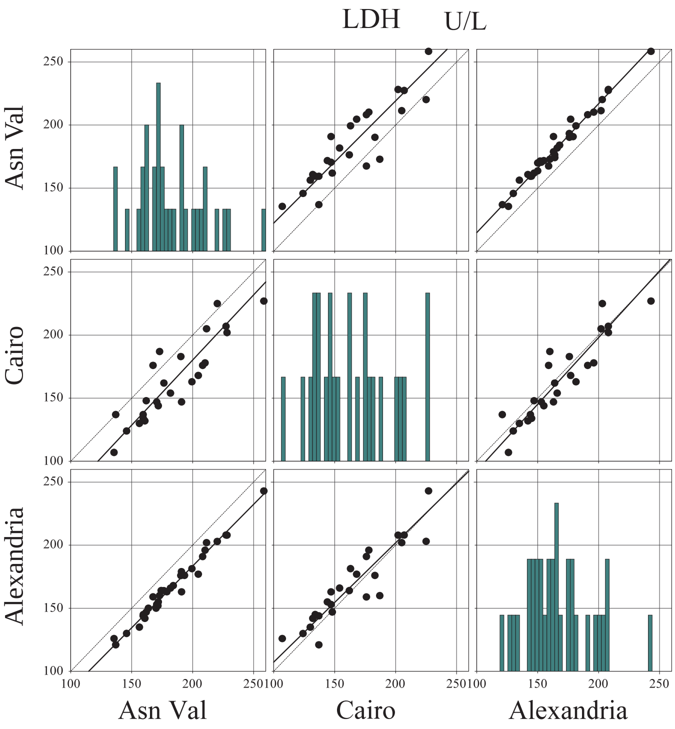
LDH U/L
Asn Val
Cairo
Alexandria
100 150 200 250

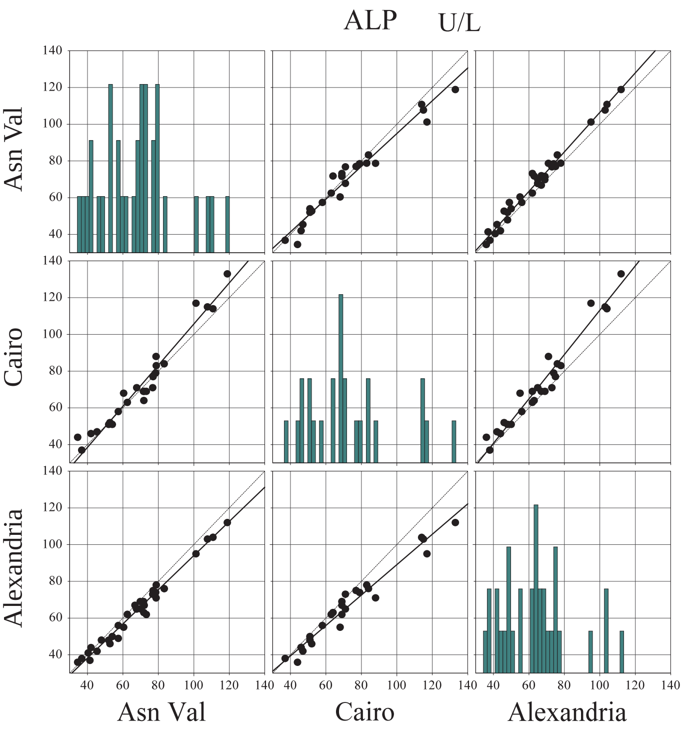
ALP U/L
Asn Val
Cairo
Alexandria
40 60 80 100 120 140

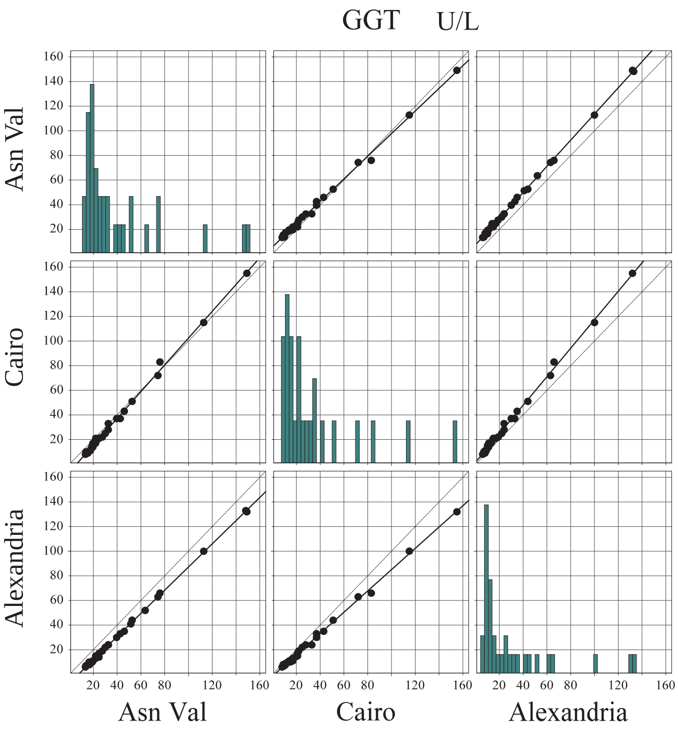
GGT U/L
Asn Val
Cairo
Alexandria
20 40 60 80 100 120 140 160

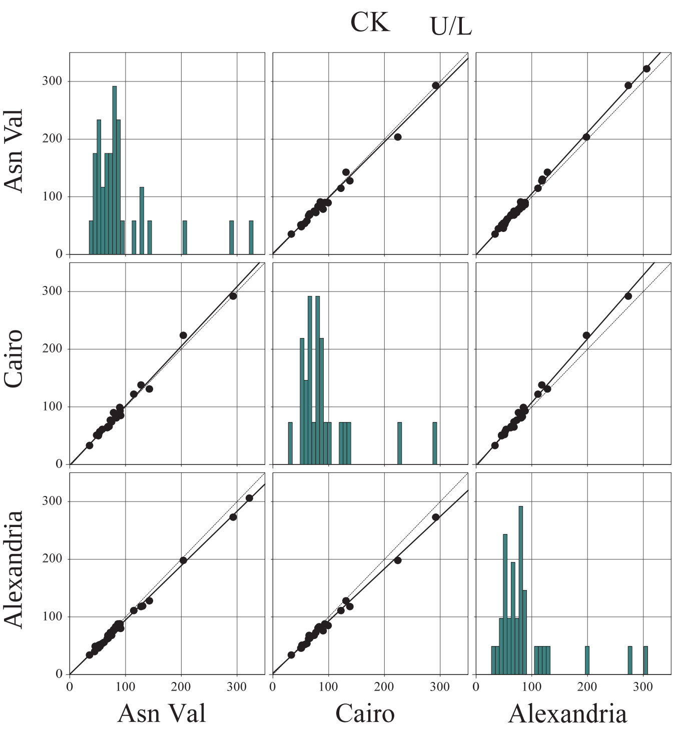
CK U/L
Asn Val
Cairo
Alexandria
0
100
200
300
Asn Val
Cairo
Alexandria

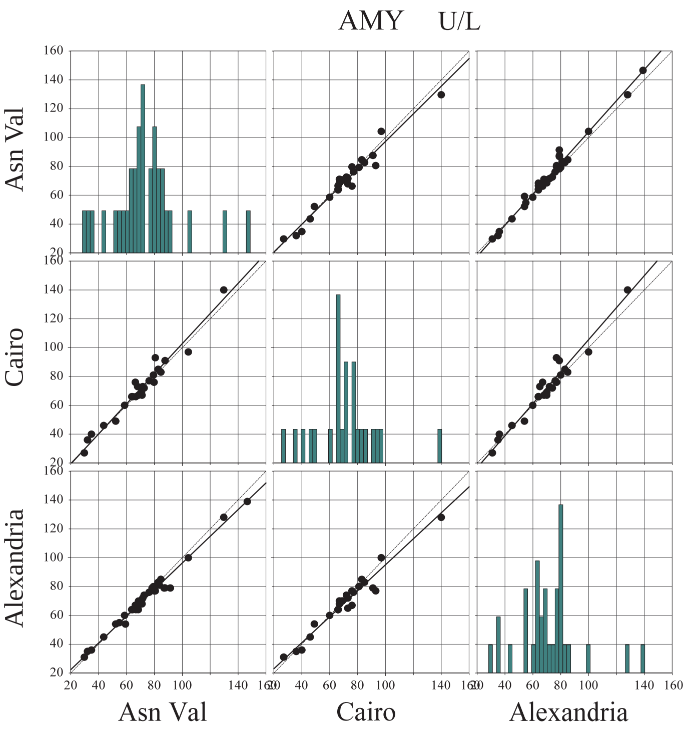
AMY U/L
Asn Val
Cairo
Alexandria
20
40
60
80
100
120
140
160
Asn Val
Cairo
Alexandria

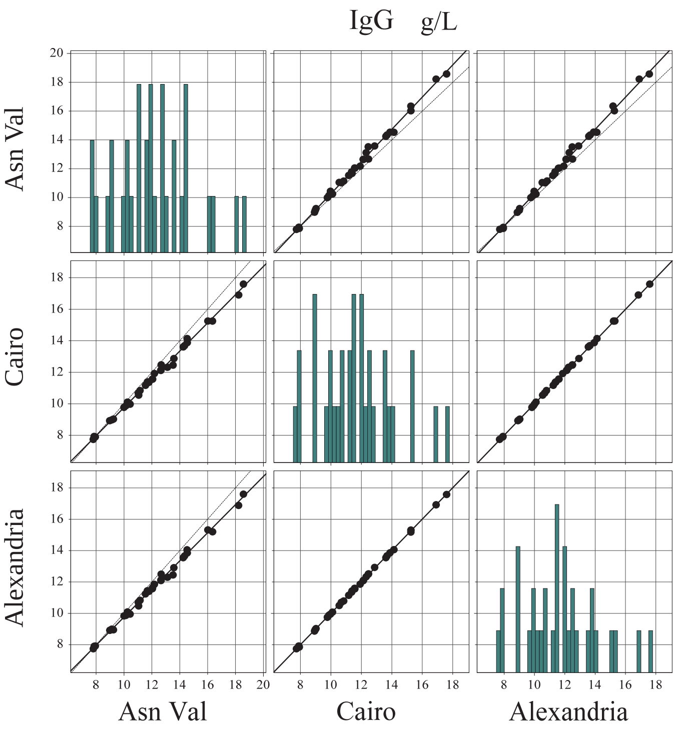
IgG g/L
Asn Val
Cairo
Alexandria
8
10
12
14
16
18
20
Asn Val
Cairo
Alexandria

IgA g/L

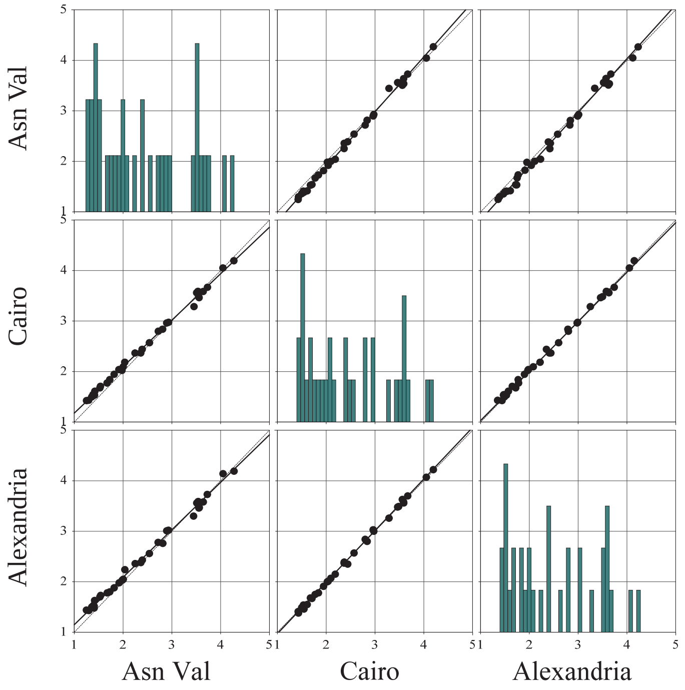

IgM g/L

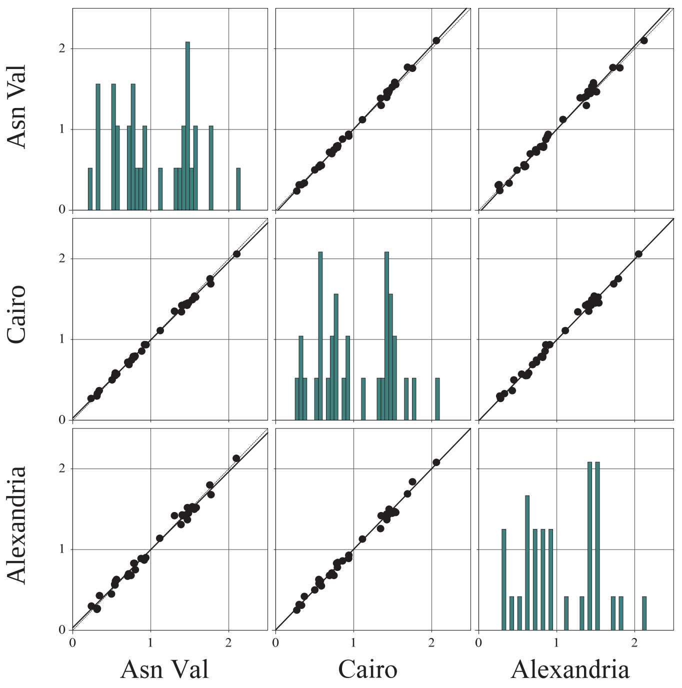

C3 g/L

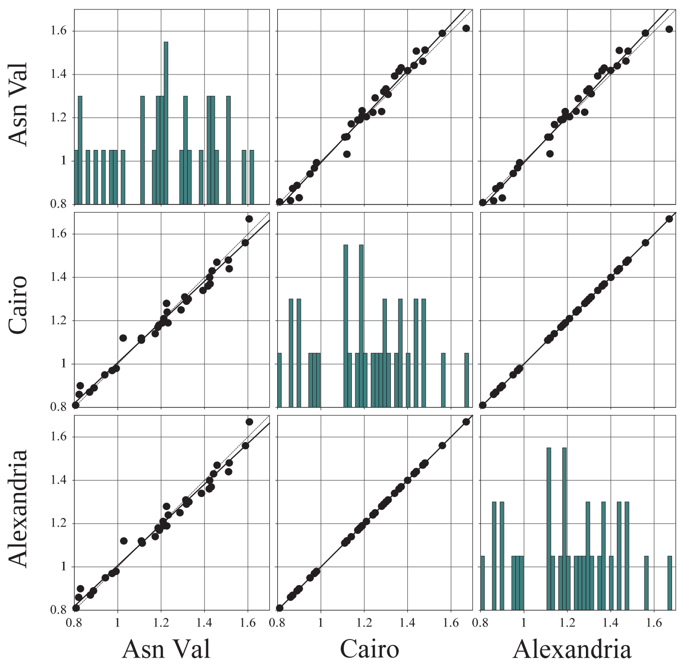

C4 g/L

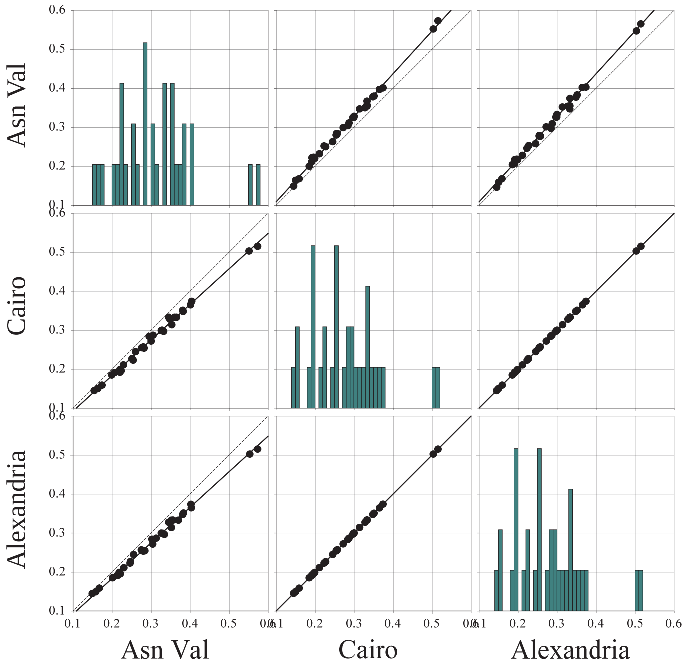

Supplement: S1 Fig — Value-assigned panel of sera were tested in Cairo and Alexandria University and their test results were compared with the assigned values (11]. The linear regression was computed by major-axis regression line. Merging of volunteers’ test results were done by aligning them to the assigned values. (PDF) [file pone.0236772.s001.pdf]
