# Supplementary material for: Establishment of reference intervals of clinical chemistry analytes for the adult population in Egypt
Source: PLoS One. 2021 Mar 19;16(3):e0236772. doi: 10.1371/journal.pone.0236772 (PMC7979267; doi:10.1371/journal.pone.0236772)
Supplement: S2 Fig — RVs were partitioned by sex (male:M, female: F) and age-subgroups (~29, 30~39, 40~49, 50~). The box in the center of each scattergram indicates the mid 50% range of RVs, and its central vertical bar represents the median. The data size is shown at the right bottom of the age group labels. Because no secondary exclusion was done for RVs, the range of the scatter plot may not match to the RI to be determined. (PDF) [file pone.0236772.s002.pdf]

Suppl. Fig 2: Sex and age-related changes in RVs of all parameters

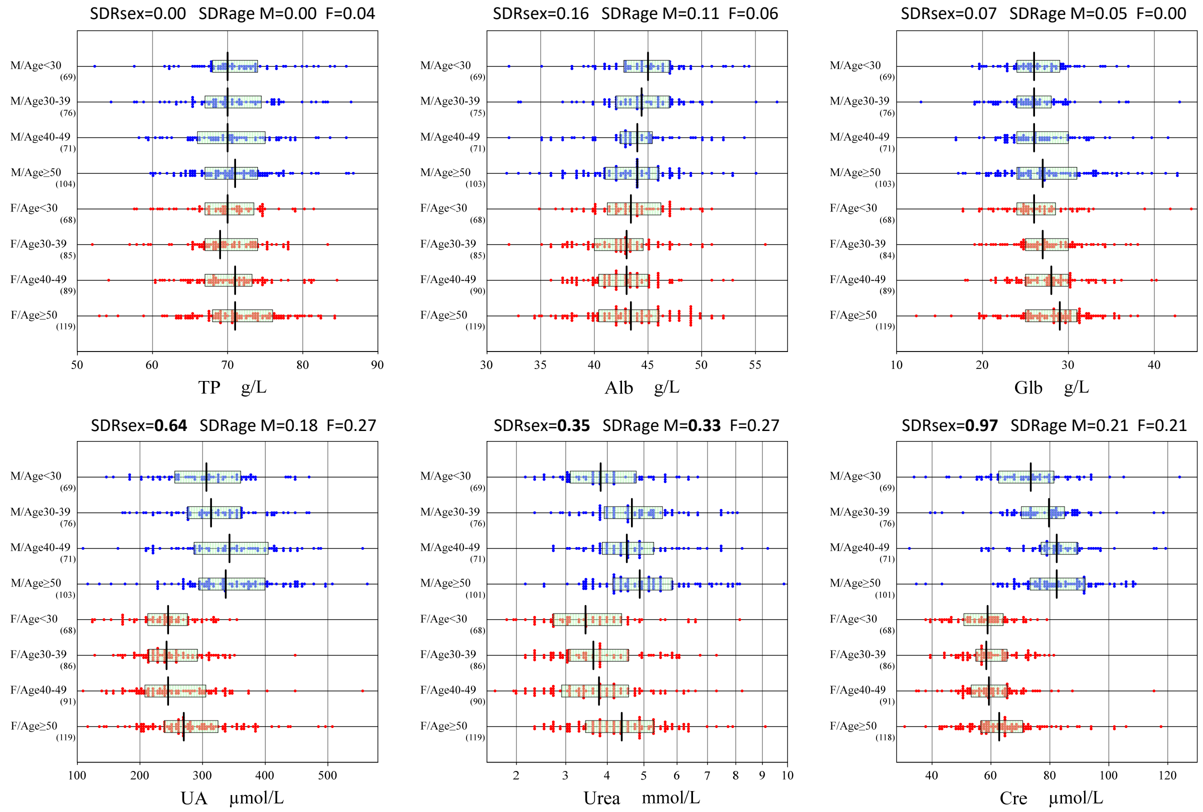

RVs were partitioned by sex (male:M, female:F) and age-subgroups (~29, 30~39, 40~49, 50~). The box in the center of each scattergram indicates the mid 50% range of RVs, and its central vertical bar represents the median. The data size is shown at the right bottom of the age group labels. Because no secondary exclusion was done for RVs, the range of the scatter plot may not match to the RI to be determined.

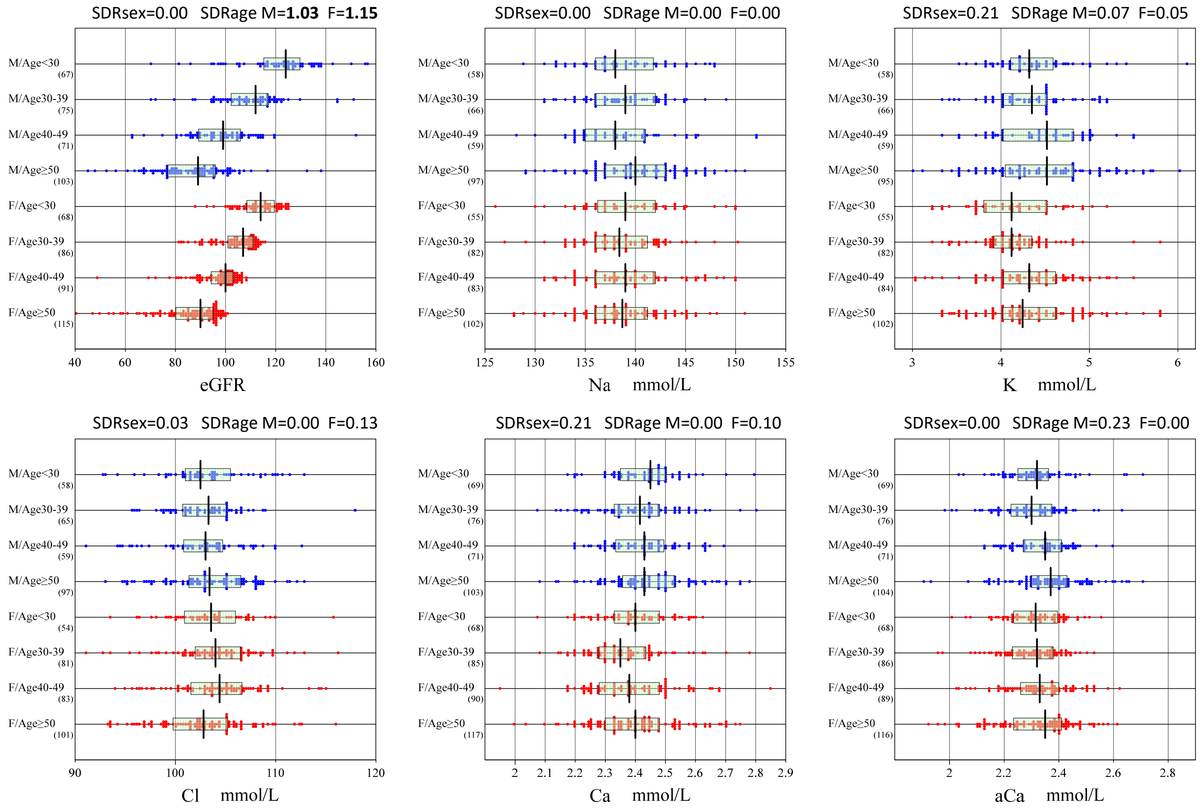

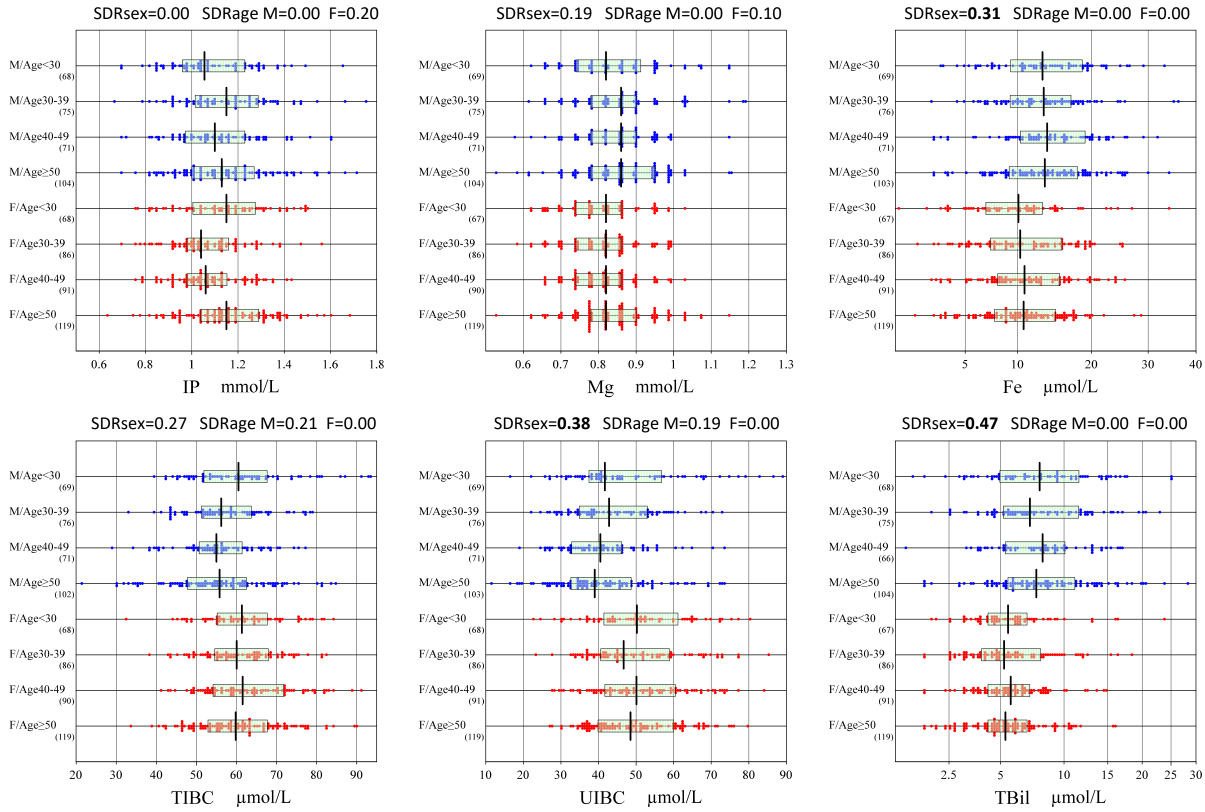
SDRsex=0.00 SDRage M=0.00 F=0.20
IP mmol/L
SDRsex=0.19 SDRage M=0.00 F=0.10
Mg mmol/L
SDRsex=0.31 SDRage M=0.00 F=0.00
Fe μmol/L
SDRsex=0.27 SDRage M=0.21 F=0.00
TIBC μmol/L
SDRsex=0.38 SDRage M=0.19 F=0.00
UIBC μmol/L
SDRsex=0.47 SDRage M=0.00 F=0.00
TBil μmol/L
M/Age<30
M/Age30-39
M/Age40-49
M/Age≥50
F/Age<30
F/Age30-39
F/Age40-49
F/Age≥50

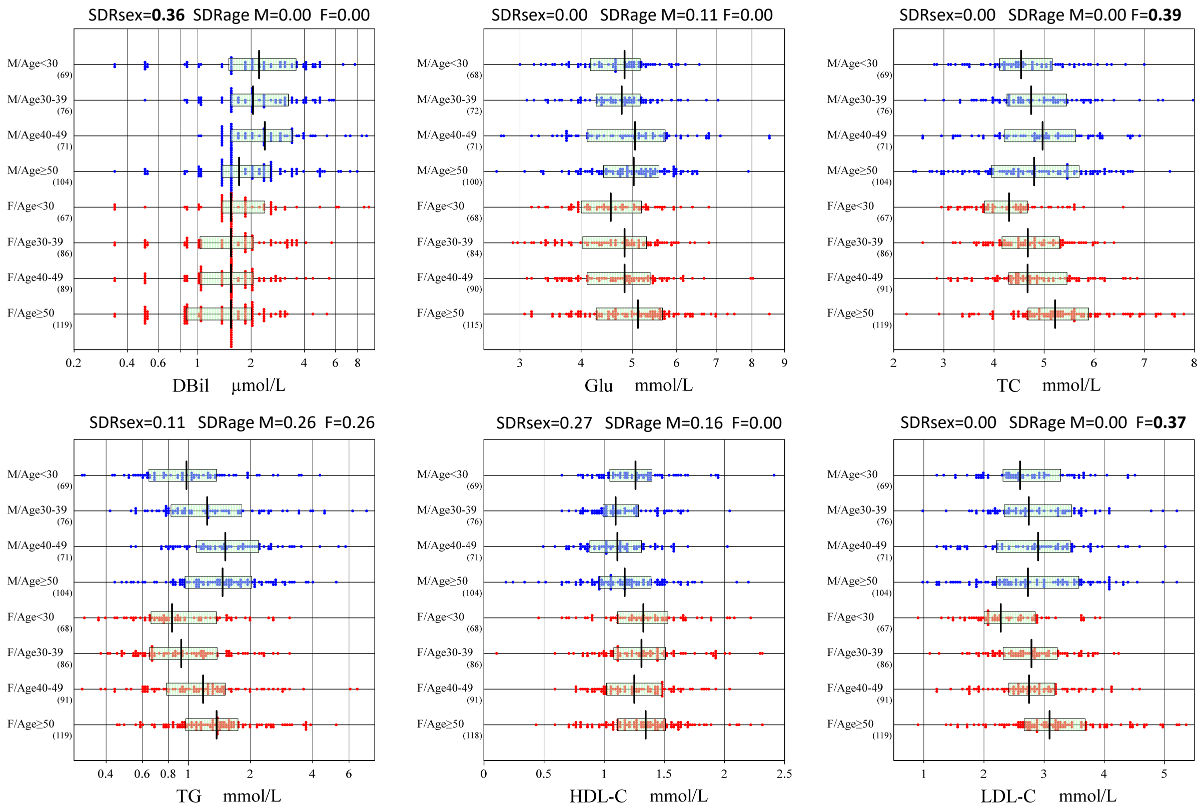
SDRsex=0.36 SDRage M=0.00 F=0.00
DBil μmol/L
SDRsex=0.00 SDRage M=0.11 F=0.00
Glu mmol/L
SDRsex=0.00 SDRage M=0.00 F=0.39
TC mmol/L
SDRsex=0.11 SDRage M=0.26 F=0.26
TG mmol/L
SDRsex=0.27 SDRage M=0.16 F=0.00
HDL-C mmol/L
SDRsex=0.00 SDRage M=0.00 F=0.37
LDL-C mmol/L
M/Age<30
M/Age30-39
M/Age40-49
M/Age≥50
F/Age<30
F/Age30-39
F/Age40-49
F/Age≥50

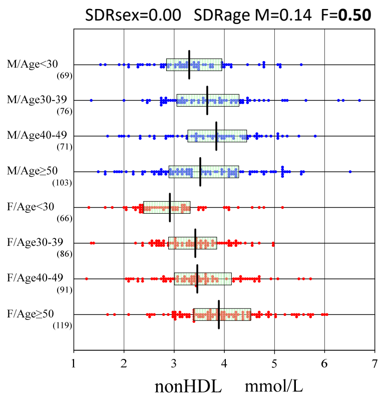
SDRsex=0.00 SDRage M=0.14 F=0.50
nonHDL mmol/L
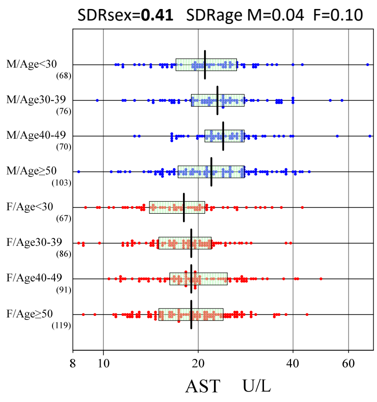
SDRsex=0.41 SDRage M=0.04 F=0.10
AST U/L
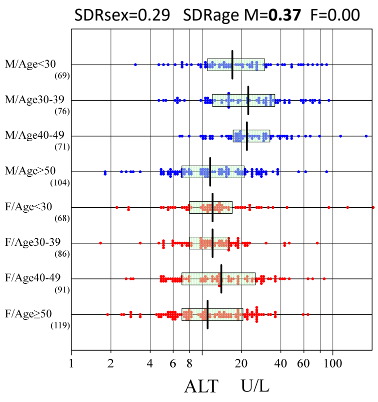
SDRsex=0.29 SDRage M=0.37 F=0.00
ALT U/L
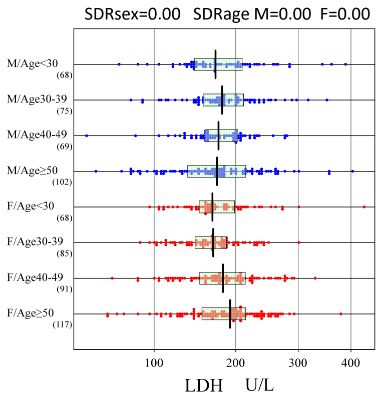
SDRsex=0.00 SDRage M=0.00 F=0.00
LDH U/L
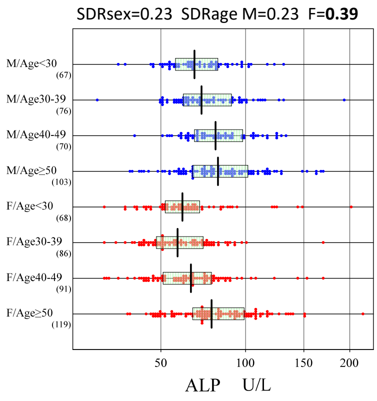
SDRsex=0.23 SDRage M=0.23 F=0.39
ALP U/L
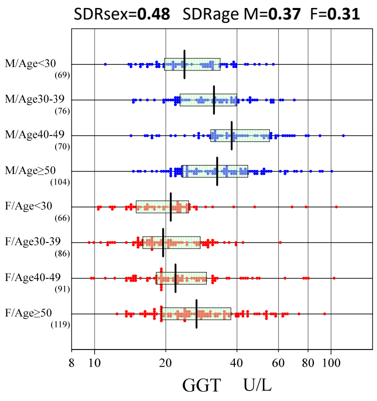
SDRsex=0.48 SDRage M=0.37 F=0.31
GGT U/L
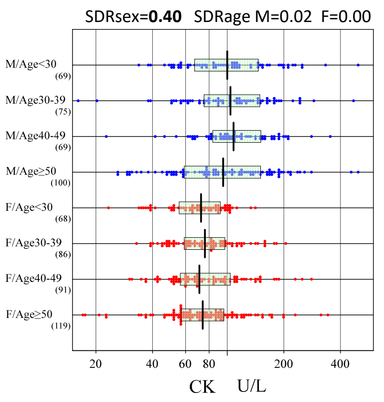
SDRsex=0.40 SDRage M=0.02 F=0.00
CK U/L
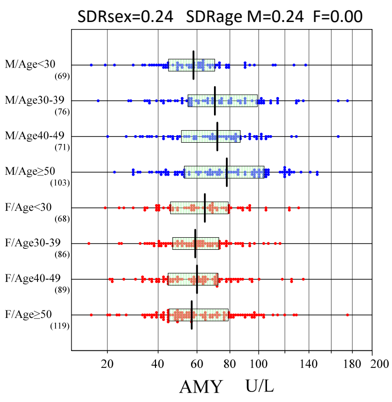
SDRsex=0.24 SDRage M=0.24 F=0.00
AMY U/L
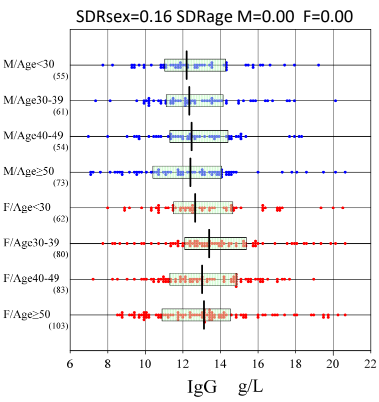
SDRsex=0.16 SDRage M=0.00 F=0.00
IgG g/L
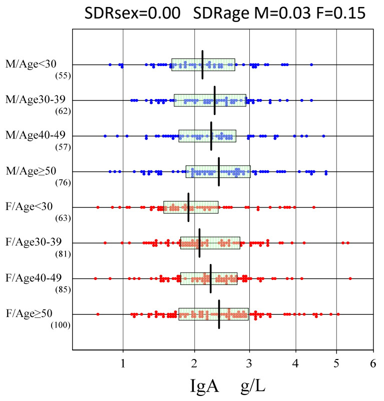
SDRsex=0.00 SDRage M=0.03 F=0.15
IgA g/L
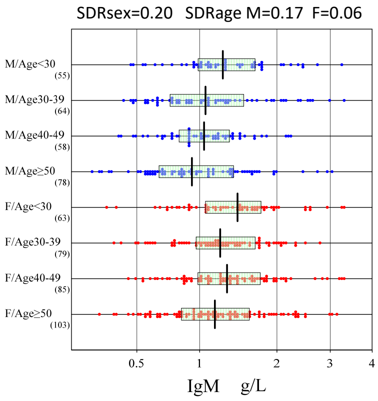
SDRsex=0.20 SDRage M=0.17 F=0.06
IgM g/L
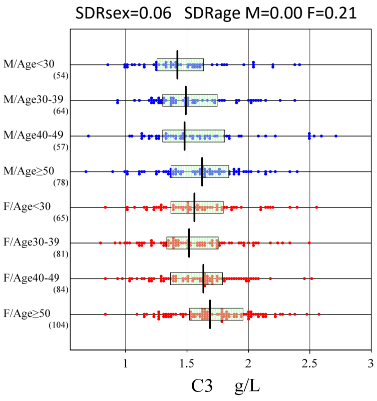
SDRsex=0.06 SDRage M=0.00 F=0.21
C3 g/L

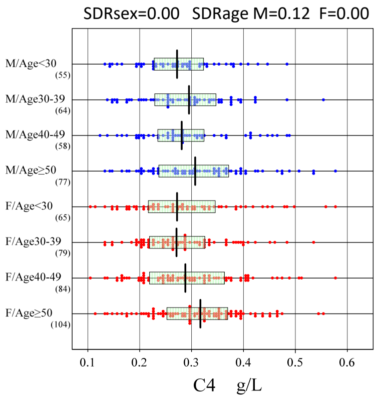
SDRsex=0.00 SDRage M=0.12 F=0.00
M/Age<30 (55)
M/Age30-39 (64)
M/Age40-49 (58)
M/Age≥50 (77)
F/Age<30 (65)
F/Age30-39 (79)
F/Age40-49 (84)
F/Age≥50 (104)
0.1 0.2 0.3 0.4 0.5 0.6
C4 g/L

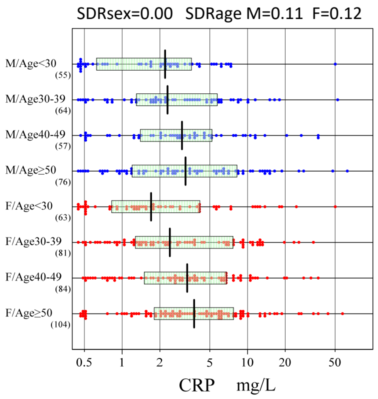
SDRsex=0.00 SDRage M=0.11 F=0.12
M/Age<30 (55)
M/Age30-39 (64)
M/Age40-49 (57)
M/Age≥50 (76)
F/Age<30 (63)
F/Age30-39 (81)
F/Age40-49 (84)
F/Age≥50 (104)
0.5 1 2 5 10 20 50
CRP mg/L

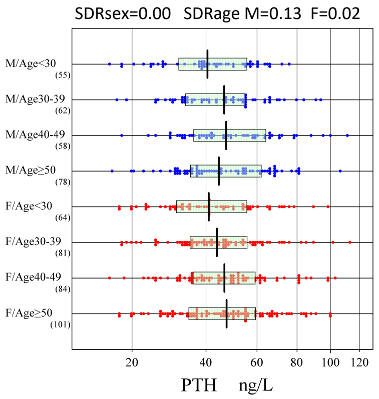
SDRsex=0.00 SDRage M=0.13 F=0.02
M/Age<30 (55)
M/Age30-39 (62)
M/Age40-49 (58)
M/Age≥50 (78)
F/Age<30 (64)
F/Age30-39 (81)
F/Age40-49 (84)
F/Age≥50 (101)
20 40 60 80 100 120
PTH ng/L

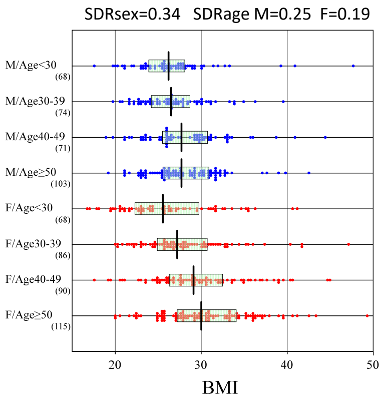
SDRsex=0.34 SDRage M=0.25 F=0.19
M/Age<30 (68)
M/Age30-39 (74)
M/Age40-49 (71)
M/Age≥50 (103)
F/Age<30 (68)
F/Age30-39 (86)
F/Age40-49 (90)
F/Age≥50 (115)
20 30 40 50
BMI

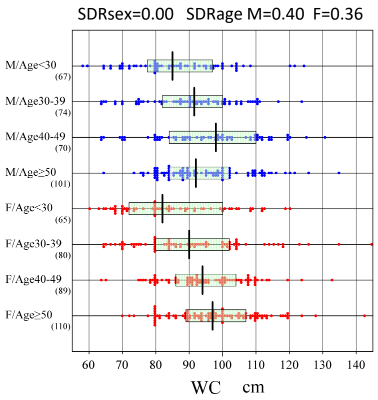
SDRsex=0.00 SDRage M=0.40 F=0.36
M/Age<30 (67)
M/Age30-39 (74)
M/Age40-49 (70)
M/Age≥50 (101)
F/Age<30 (65)
F/Age30-39 (80)
F/Age40-49 (89)
F/Age≥50 (110)
60 70 80 90 100 110 120 130 140
WC cm

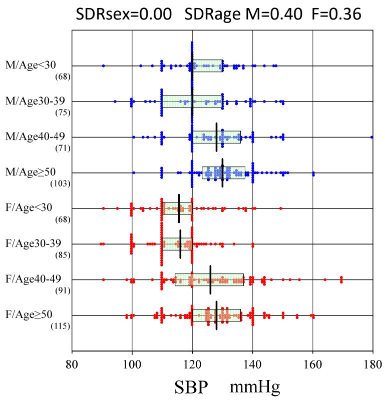
SDRsex=0.00 SDRage M=0.40 F=0.36
M/Age<30 (68)
M/Age30-39 (75)
M/Age40-49 (71)
M/Age≥50 (103)
F/Age<30 (68)
F/Age30-39 (85)
F/Age40-49 (91)
F/Age≥50 (115)
80 100 120 140 160 180
SBP mmHg

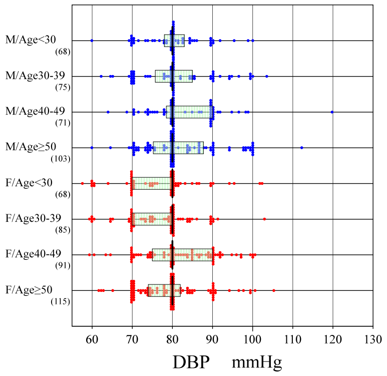
M/Age<30 (68)
M/Age30-39 (75)
M/Age40-49 (71)
M/Age≥50 (103)
F/Age<30 (68)
F/Age30-39 (85)
F/Age40-49 (91)
F/Age≥50 (115)
60 70 80 90 100 110 120 130
DBP mmHg

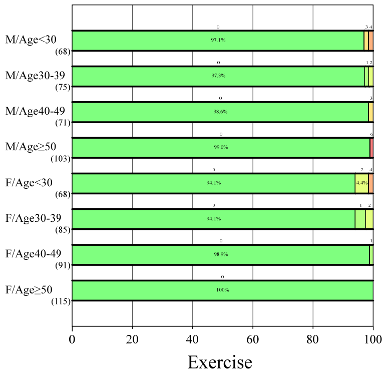
M/Age<30 (68)
M/Age30-39 (75)
M/Age40-49 (71)
M/Age≥50 (103)
F/Age<30 (68)
F/Age30-39 (85)
F/Age40-49 (91)
F/Age≥50 (115)
0 20 40 60 80 100
Exercise

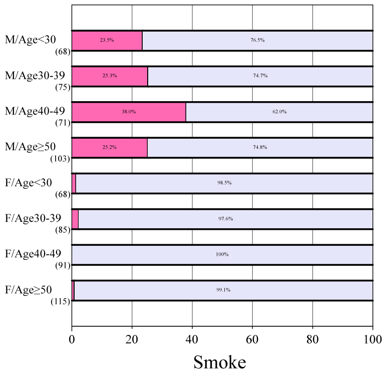
M/Age<30 (68)
M/Age30-39 (75)
M/Age40-49 (71)
M/Age≥50 (103)
F/Age<30 (68)
F/Age30-39 (85)
F/Age40-49 (91)
F/Age≥50 (115)
0 20 40 60 80 100
Smoke

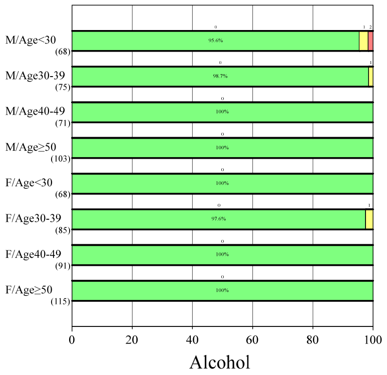
M/Age<30 (68)
M/Age30-39 (75)
M/Age40-49 (71)
M/Age≥50 (103)
F/Age<30 (68)
F/Age30-39 (85)
F/Age40-49 (91)
F/Age≥50 (115)
0 20 40 60 80 100
Alcohol

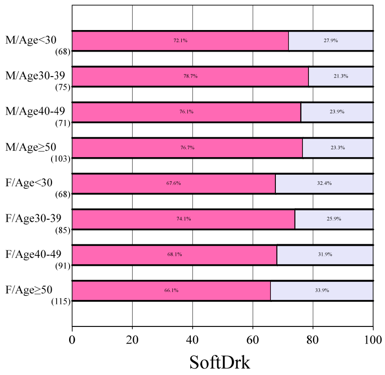
M/Age<30 (68)
M/Age30-39 (75)
M/Age40-49 (71)
M/Age≥50 (103)
F/Age<30 (68)
F/Age30-39 (85)
F/Age40-49 (91)
F/Age≥50 (115)
0 20 40 60 80 100
SoftDrk
