# Supplementary figures and images for: Establishment of reference intervals of clinical chemistry analytes for the adult population in Egypt
Source: PLoS One. 2021 Mar 19;16(3):e0236772. doi: 10.1371/journal.pone.0236772 (PMC7979267; doi:10.1371/journal.pone.0236772)

Suppl. Fig 3: Between-region-differences in RVs observed in 5 analytes

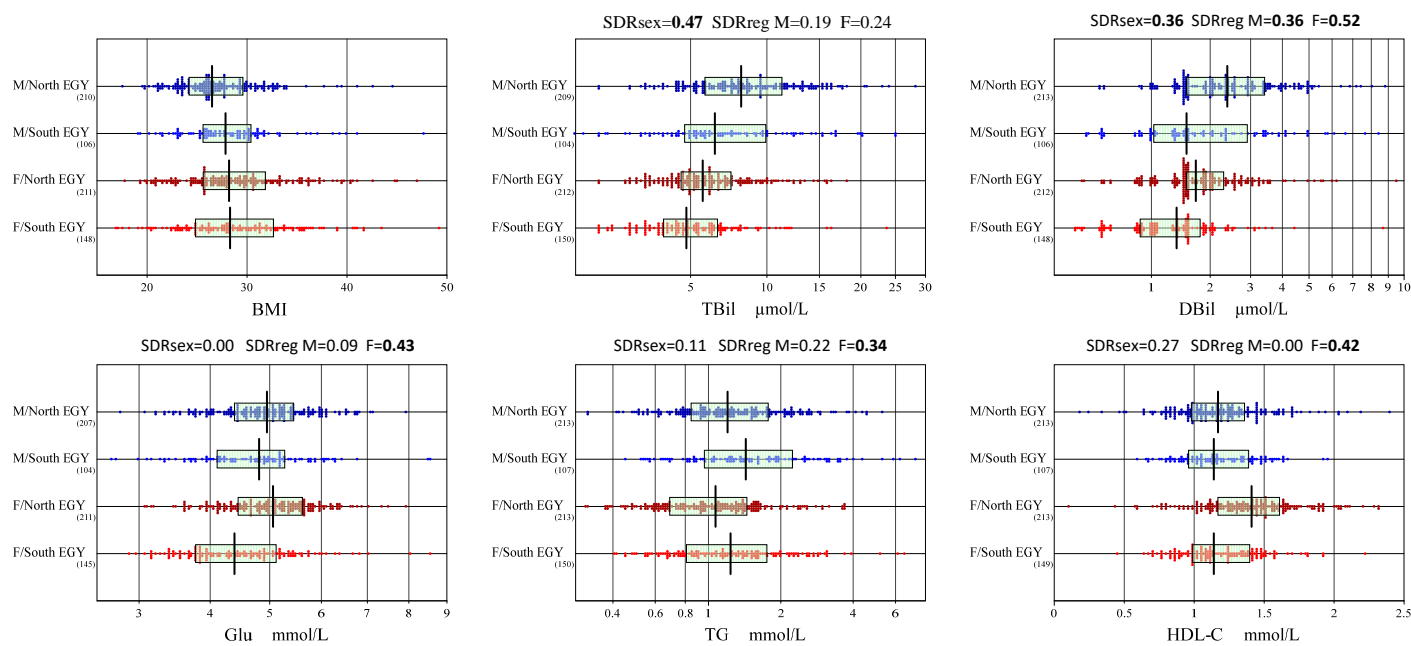

Supplement: S3 Fig — RVs of five analytes, TBil, DBil, Glu, TG, and LDL-C that showed high SDR for between-region differences (SDRreg) were partitioned into four groups by sex and region (North vs. South Egypt). Values of BMI were also shown subgrouped by sex and region to prove that the regionality is independent of the levels of BMI. (PDF) [file pone.0236772.s003.pdf]
