# Supplementary material for: Establishment of reference intervals of clinical chemistry analytes for the adult population in Egypt
Source: PLoS One. 2021 Mar 19;16(3):e0236772. doi: 10.1371/journal.pone.0236772 (PMC7979267; doi:10.1371/journal.pone.0236772)
Supplement: Attachment — Submitted filename: reviewer comments PONE-D-20-21548.pdf [file pone.0236772.s009.pdf]

Editor in Chief, PLOS ONE

Establishment of Reference Intervals of Clinical Chemistry Analytes for the Adult Population in Egypt

PONE-D-20-21548

Reviewer(s)' Comments to Author:

Reviewer:

Comments to the Author

The manuscript established the reference interval for 34 major chemistry analytes and discussed the sources of variation. The study is part of the global reference interval study of the IFCC. The manuscript has many details about data analysis but some core aspects have weakened the manuscript which need to be answered and corrected.

Specific comments

Manuscript:

Material and methods

1. Study design

- The study was performed in two different institutes. Therefore, the ethical approval number should be unified but it seems Cairo University ethical approval number only has been mentioned. What about Medical Research Institute of Alexandria University was there any specific ethical approval number? If yes then please mention it.

2. Recruitment of reference individuals

"A total of 691 apparently healthy Egyptian volunteers were recruited from Cairo, Alexandria, Delta, Suez Canal, and Upper Egypt; Fayoum, Beni-Sueif or areas southern as faras Nubia". It is not so clear if samples were collected from volunteers from each of these locations or volunteers came to one cite for samples to be collected.

4. Analytes and measurements

- Page 2: Suppl table 1: does not show the formulae for the calculated parameters Globulins (Glb), estimated glomerular filtration rate (eGFR), adjusted calcium (aCa), total iron binding capacity (TIBC), and non-high-density lipoprotein (nonHDL).

- PTH is the only anlyte based on immunoassay measurement. Why it has been included in this study in particular while the study is dedicated to chemistry tests?

- PTH can be affected by calcium and vitamin D intake, did the author consider them when RI for PTH has been derived (as exclusion criteria). The same thing for Iron. If yes, then how many subjects were excluded?

- When the study was started (month/year) and when it was ended (month/year)?

- Page 3: IgG, IgM, IgA, C3, C4, CRP and PTH were measured 8 months after the collection, why and how their stability was assessed?

5. Between-center method comparison and standardization

- A panel composed of 50 healthy volunteers' sera with assigned values for 34 chemistry and immunoturbidimetry analytes was measured by the two different centers for merging and standardization of test results. How this panel of samples was transferred physically from one center to another? And how their stability was guaranteed?

- A summarized table is needed to show number of samples, BMI, sex distribution, age, BP and other anthropometric measurements for each cite and in total.

- In this study, samples were analyzed in two independent labs. The panel was used to standardize test results between both cites. In other involved countries in the global study all samples were sent to the main lab for analysis and a panel was used to standardize results between different labs. Clarify if the protocol followed in this study contradicts the harmonized protocol created by the C-RIDL or not.

- For between-day quality control, no data are shown.

Results:

- Page 6: The head title in Table 1 "South Egy" to be changes to "Region".

- Table 3: TBil and DBil unit mmol/L to be corrected
  TP, Alb, Glb unit g/L to be corrected in RI in conv, unit.

- Page 9: Supp Fig 3 shows low glucose, DBil and TBil for samples from South Egypt. In addition to this in Figure 2, TBil is the lowest compared to other countries (Glu is not included in the figure). It seems these outcomes are not compatible with the prevalence and rates of diabetes and hepatitis in Egypt compared to other countries.
  Did the author investigate the possibility of the presence of any gap in the followed C-RIDL protocol regarding samples integrity i.e. during samples collection, transportation, delay in separation, sun exposure etc. before samples were analysed.

Discussion:

- Page 12: "Mean BMI in our study was 28.2±5.0" the unit needs to be added
